# Evaluating the Cognition, Behavior, and Social Profile of an Adolescent With Learning Disabilities and Assessing the Effectiveness of an Individualized Educational Program

**Published:** 2014

**Authors:** Preeti Tabitha Louis, Isaac Arnold Emerson

**Affiliations:** 1 Assistant Professor, School of Social Sciences and Languages, VIT University, Vellore, Tamil Nadu, India.; 2 Associate Professor, School of Bio Sciences and Technology, VIT University, Vellore, Tamil Nadu, India.

**Keywords:** Adolescence, Co-therapists, Disability, Individualized Educational Program

## Abstract

**Objective:** The present study seeks to outline a holistic assessment method that was used in understanding problems experienced by an adolescent boy and in designing and implementing an individualized educational program.

**Methods:** An adolescent child referred for concerns in learning was screened for learning disability using standardized inventories and test batteries. The Connors Parent and Teacher Rating Scales (short forms), Wechsler's Intelligence Scale for Children (WISC), the Vineland Social Maturity Scale (VSMS), and the Kinetic Family Drawing (KFD) test were used to assess the behavior, cognition, and social profile of the child. An individualized educational program was designed and this intervention was provided for 6 months by using parents as co-therapists. Participant and parent interview schedules were used in identifying underlying issues of concern. The child was reassessed 6 months after the intervention was provided.

**Results: **Findings on the Connors Parent Rating Scale revealed scores that were greater than the 50^th^ percentile on the domains of inattention and cognitive problems. On the Connors Teacher Rating Scale, we observed scores greater than the 50^th^ percentile on the hyperactivity, cognitive problems, and the inattention domains. The WISC revealed that the child had a "Dull Normal" Intellectual functioning and there was also a deficit of 2 years on the social skills as tested by the Vineland Social Maturity Scale (VSMS). The Kinetic Family Drawing Test revealed negative emotions within the child. Post intervention, we noticed a remarkable improvement in the scores across all domains of behavior, social, and cognitive functioning.

**Conclusion:** Designing an individualized education program that is tailored to the specific needs of the child and using parents as co-therapists proved to be an effective intervention.

## Introduction

Learning disability is best described as a disorder in which one or more of the basic psychological processes involved in understanding or in using language, spoken or written, manifests itself as an imperfect ability to listen, think, speak, read, write, spell, or do mathematical calculations (1). This includes conditions such as perceptual disabilities, brain injuries, minimal brain dysfunctions, dyslexia, and developmental aphasias (2). However, it does not include learning disabilities that are primarily the result of visual, hearing, or motor disabilities, mental retardation, emotional disturbance, or environmental, cultural, or economic disadvantages according to the Individuals with Disabilities Education Act formulated in 1999 (3).

Learning disabilities fall into broad categories based on four stages of information processing that are used in learning; input, integration, storage, and output (4). The most common learning disability is developmental dyslexia that accounts for 5-10% of a given population although there have been no studies to indicate an accurate percentage (5). Dyslexia is characterized by deficits in reading and is most often termed as a reading disability. This can affect any part of the reading process (including difficulty with accurate or fluent word recognition, or both), word decoding, reading rate, prosody (oral reading with expression), and reading comprehension. Before the term "dyslexia" came to prominence, it was known as "word blindness" (6). 

Common indicators of reading disability include difficulty with phonemic awareness (the ability to break up words into their component sounds) and difficulty with matching letter combinations to specific sounds also known as sound-symbol correspondence (7). Impaired written language ability may include impairments in handwriting, spelling, organization of ideas, and composition. This term was used as an overarching term for all disorders of written expression. Others, such as the International Dyslexia Association, have used the term "dysgraphia" exclusively to refer to difficulties with handwriting only (8). 

Sometimes called dyscalculia, a math disability can cause such difficulties as learning math concepts, such as quantity, place value, and in understanding the concept of time (9). It may also characterize difficulties in memorizing math facts, difficulty organizing numbers, and understanding how problems are organized on a page. Dyscalculic children are often referred to as having poor "number sense" (10). There can be another type of learning disability called the nonverbal learning disability. This often manifests as motor clumsiness, poor visual-spatial skills, problematic social relationships, difficulty with math, and poor organizational skills. These individuals often have specific strengths in the verbal domains, including early speech, large vocabulary, early reading and spelling skills, excellent rote-memory and auditory retention, and eloquent self-expression (11).

Learning disabilities are often identified by school psychologists, clinical psychologists, and neuropsychologists through a combination of intelligence testing, academic achievement testing, classroom performance, social interaction, and aptitude testing (12). Other areas of assessment may include perception, cognition, memory, attention, and language abilities. The resulting information is used to determine whether a child's academic performance coincides with his or her cognitive ability. The most commonly used comprehensive achievement tests include the Woodcock-Johnson III (WJ III), Wechsler Individual Achievement Test III (WIAT III), the Wide Range Achievement Test 4 (WRAT 4), and the Stanford Achievement Test–10^th^ edition. These tests include measures of many academic domains that are reliable in identifying areas of difficulty (13).

Currently, almost 2.9 million school-aged children in the United States are classified as having specific learning disabilities and receive some kind of special education support (14). In fact, over half of all children who receive special education have a learning disability (24^th^ Annual Report to Congress, 2002). 

In India 12-13% of children are identified as having learning disability (15). However, during the last decade, awareness about these children's behavioral, social, and emotional problems have considerably increased. This awareness was demonstrated by a meta-analysis that explored the relation between learning disability and behavioral problems (16). Furthermore, there were also other significant researches that explored the relationship between learning disability and social skill deficits (17). Both of these studies provided convincing evidence that children and adolescents with learning issues experience social problems such as low self-esteem, emotional difficulties like depression, and also conduct problems such as aggression.

Past investigations of children with learning disabilities had focused mainly on children's cognitive and learning problems and on interventions designed to ameliorate these problems, but currently research has focused on a treatment-oriented diagnostic process known as response to intervention (RTI) (18). This includes early screening for children, placing those who are having difficulty into research-based early intervention programs, rather than waiting until they meet diagnostic criteria (19). Their performance can be closely monitored to determine whether increasingly intense intervention results in adequate progress. Those who respond will not require further intervention (20).

Early remediation can also greatly reduce the number of children meeting diagnostic criteria for learning disabilities. Focus on learning disabilities and the provision of accommodations in school fail to acknowledge that people have a range of strengths and weaknesses, and places undue emphasis on academic success by insisting that people should receive additional support in this arena but not in music or sports (21). 

Today, we use the individualized education program (IEP) that is tailored to the individual student's needs as identified by the evaluation process (22). This helps teachers and related service providers understand the student's disability and how the disability affects the learning process. The IEP also describes how the student learns, how the student best demonstrates that learning and what teachers and service providers will do to help the student learn more effectively.

In this study, we explored the cognitive, behavioral, and social profile of an adolescent boy and also determined the impact of learning disability on the family by the use of interview schedules. Parent, child, and teacher reports were used to identify issues of concern that hindered the child from coping well at home and school. The focus of the study was to draw attention to the use of an individualized education program (IEP) which was designed by the professional team using parents as co-therapists. The effect of the intervention was verified by reassessing the child six months after the therapeutic intervention.

## Materials and Methods

***Setting***

The present study is a case description of the assessment methods used for an adolescent child with learning disability. He was referred to the Developmental Pediatrics Department of the Christian Medical College and Hospital, Tamil Nadu, India, for concerns in reading, writing, and arithmetic. Upon examination by the pediatrician, he was diagnosed with learning disability and was referred to the psychologist for further evaluations. As a detailed assessment was necessary, voluntary consent was obtained from the parents, child, and also from the child's teacher to participate in the study. They were reassured that personal interviews with the child, parent, or the teacher will be kept confidential and for this purpose a one-way-mirror room was used. All the assessments were done in the clinical setting by the psychologist working at the Developmental Pediatrics Department of the hospital both pre and post intervention.

***Participants***

The study was conducted on a 13 year old adolescent boy who was a single child with no siblings belonging to a middle-class North Indian family at Manali, India. The child was studying in the 8^th^ grade at a private school at Manali. The inclusion criteria adopted for this study are that the child must present with concerns in reading, writing, and arithmetic, and the age of the child must be between 13-18 years. The child should also fulfill the criteria for learning disability according to the DSM IV-TR in axis I as diagnosed by the pediatrician. The child must be referred for a psychological assessment to screen for learning disability and to implement an intervention. There should be no other previous history of interventions used for the child. The exclusion criteria adopted are that there must be no indications of personality, mood, or anxiety disorders in the child, and that there should be no other comorbidities such as hearing loss or neurological impairments. 

***Procedures***

The screening for learning disability was performed by the psychologist of the Developmental Pediatrics Department pre and post intervention and comprised of 6 sessions each. Testing of intelligence, behavior, and the social skills were performed in 3 sessions and interviewing the child and parents in 2 sessions. Scheduled interviews and careful participant observation were used as part of the assessment. Discussion on the individualized educational program (IEP), which was the intervention that focused on implementing assistive techniques for improving academic performance, was performed in 1 session. The IEP was planned by the psychologist after consulting and coordinating with the concerned pediatrician, and occupational and speech therapists who also spent time with the child and his family. They provided intervention strategies to enhance writing and communication skills. The standardized tests included the Wechsler Intelligence Scale for Children (WISC-IV) which was used to understand the cognitive level of the child, The Vineland Social Maturity Scale (VSMS) which was administered to determine the social age and social quotient of the child, and The Connors Parent and Teacher Rating Scales (3^rd^ edition-short form) that was used to determine the behavioral profile of the child. Projective tests such as the Kinetic Family Drawing Test (KFD) were administered to understand underlying family issues and concerns within the child. In addition to these tests, the Child and Adolescent Sleep Checklist (CASC-student version), the Adolescent Food Habit Checklist (AFHC), and the Mood and Feelings Questionnaire (MFQ) were also used. Brief scheduled interviews with the child, parents, and the teacher were conducted to obtain information regarding the behavior of the child at home and school. Scores and test results were discussed with the parents to help them comprehend their child's needs and concerns. 

The design of the individualized education program (IEP) was tailored to the specific needs of the child and parents were instrumental in providing feedback and in planning the intervention. The teacher too played a vital role in following the suggestions in school and in keeping the professional team informed of the progress made. The school was approached for making reforms in accommodating the changes that the child needed in his learning style. Suggestions on improving his communication, social skills, and self- esteem were also included in the IEP. 

To develop reading and listening skills, we encouraged the family to spend time conversing and reading short stories to him. They could motivate him to develop reading habit by buying him short and easy to read story books. They could also read the headlines of the newspaper to him, which may help introduce him to new words and sentence styles. He could begin by reading aloud alone and then in front of his parents, and gradually he could be trained to read to his peers. This will enable him to cope with his shyness and reading anxiety. 

To develop his vocabulary skills, we encouraged the family to introduce new words to him every day. They could do this whenever they went shopping or when they visited the market or when they were driving home from school. Words that could be taught along with pleasurable experiences would be retained for a longer time. They could also positively reinforce the child with encouraging compliments whenever he made an attempt to use those words. Since the child found it difficult to narrate experiences, we encouraged the family to allow the child to look at a picture and then describe slowly what he saw in the picture. This would help the child improve in his speaking and communication skills. He could also listen to a short story on an audiotape and then try to narrate the story to his mother or father. This will improve his ability to remember and recall information. 

We discussed with the parents whether they could consider the use of assistive technology in helping him learn. The child could watch useful documentaries and animated videos on science, English, and arithmetic. This would instill interest and also make learning easier and interesting for him. We suggested the use of highlighters and special glitter pens while reading to improve visual attention and concentration. He could also audiotape the lessons that the teacher taught in school and listen to them at leisure at home. This would greatly reduce the stress at school and help the child learn at his own pace. We suggested the use of computers to reduce the burden on writing. Since the child found it difficult to complete his homework or to copy large texts of information, using the computer may help reduce writing stress and also enhance his creativity. The child could type his answers instead of using the notebook and hence avoid fatigue and messy work.

Experience based learning was suggested for the understanding of concepts in arithmetic. He could play shopping class at home to understand the concept of money. His parents could give him currency and then teach him calculations and simple transactions with it. Every hour, he could practice by telling the time and his parents could help him understand simple calculations by asking him to go to the shop and purchase items such as a soap or oil. This would help the child understand and execute simple mathematical calculations mentally. They could also stick arithmetic number tables beside his bed so that he could revise them before going to sleep. 

Peer learning at school was suggested to increase social skills and to instill self-confidence in the child. The teacher could help by asking a few children to sit together after school hours and help one another discuss and review homework and assignments. This would not only help children in gaining better understanding of academic work, but would also promote healthy relationships. The child would benefit from this experience by gaining self-acceptance and feeling more secure in the classroom. We discussed ways to reduce the tuition hours and to enable the teacher to become more involved in helping the child by providing simple worksheets of the lesson taught every day. Tuitions were only adding to the burden of what was already there, demanding more work and time from the child. Providing worksheets would simplify the lesson and the child could be made to focus on only what was necessary to be learnt. The teacher could be more accommodative by checking his homework whenever he could finish it during the week, and not stressing him too much with deadlines.

The family was encouraged to give the child small responsibilities at home and at school such as watering the garden, feeding his pet, or cleaning the house. This would help the child appreciate himself and to see that he too can do things just like other children. Appropriate reinforcements could be chosen so as to motivate him such as a verbal praise, a surprise visit to a movie theatre, or buying him his favorite food once a week. This would enable the child to be competent and to seek more challenges. Parents were asked to spend more time with the child talking and listening to him. The father was encouraged to spend time in play activities and the mother too was told to focus on the child's strengths and not on his inadequacies. The parents were asked to be supportive and to understand their child and not to be demanding. All of these suggestions were to be executed for 6 months after which the child would be reassessed.

***Measures***

*The* *Wechsler Intelligence Scale for Children* *(* *WISC* *-IV)*

This test was developed by Dr. David Wechsler and is an individually administered intelligence test for children between the ages of 6 and 16 years (23). The WISC-IV takes 65-80 minutes to administer and generates an IQ score which represents a child’s general cognitive ability. There are four indexes namely the verbal comprehension, perceptual reasoning, working memory, and the processing speed index. The full scale IQ ranges from lowest 40 to highest 160 points. Subtests are given for additional examination of processing abilities. The verbal comprehension index assesses children's ability to listen to a question, draw upon learned information from both formal and informal education, reason through an answer, and express their thoughts aloud. It can tap preferences for verbal information, a difficulty with novel and unexpected situations, or a desire for more time to process information rather than to decide on the spot. This index is a good predictor of readiness for school and achievement orientation, but can be influenced by background, education, and cultural opportunities. The perceptual reasoning index assesses the child's ability to examine a problem, draw upon visual-motor and visual-spatial skills, organize their thoughts, create solutions, and then test them. It also taps preferences for visual information, comfort with novel and unexpected situations, or a preference to learn by doing. The working memory index assesses the child’s ability to memorize new information, hold it in short-term memory, concentrate, and manipulate that information to produce some result or reasoning processes. This is important in higher-order thinking, learning, and achievement and this taps concentration, planning ability, cognitive flexibility, and sequencing skill, but is also sensitive to anxiety. It is an important component of learning and achievement, and the ability to self-monitor. Finally the processing speed index assesses the abilities of focusing attention and quickly scanning, discriminating, and sequentially ordering visual information. It requires persistence and planning ability, but is sensitive to motivation, difficulty working under time pressure, and motor coordination. Cultural factors seem to have little impact on it. This test is also related to reading performance and development. The average reliabilities of WISC-IV fall between 0.78 and 0.88, suggesting that there is a reasonable amount of score consistency. The test retest reliabilities yielded correlations in the 0.80s and 0.90s for both subtest scores and composite scores, suggesting good test retest reliability. Content validity was established by reviewers and experts by creating content similar to other established tests to expand the evaluation base of the WISC IV and correlations were found to range from 0.83 to 0.89. Discriminant validity was established by conducting an updated factor analysis. Each of the subtests on an index has factor loadings above 0.60 with their own index. For convergent validity, correlations between the WISC IV and other Wechsler tests were found to be between 0.6 and 0.8 that was most appropriate.

*The Vineland Social Maturity Scale (VSMS)*

An Indian adaptation of the Vineland Social Maturity Scale was used to assess children aged 0-16 years in the areas of self-help general, self-help dressing, self-help eating, self-direction, locomotion, communication, occupation, and socialization (24). The scale yields a social age and a social quotient, which can be considered an approximate intelligence quotient. This test is found to have a correlation of 0.85 to 0.96 with the Stanford-Binet Intelligence Scale.

*The Connors Parent Rating Scale (CPRS-3* ^rd^ *edition-short form)*

This instrument is used for routine screenings in schools, mental health clinics, residential treatment centers, pediatric offices, juvenile detention facilities, child protective agencies, and outpatient settings (25). The CPRS can help in measuring hyperactivity in children and adolescents through routine screening, providing a perspective of the child’s behavior from those who interact with the child on a daily basis and in establishing a base point prior to beginning therapy and to monitor treatment effectiveness and changes over time. It provides valuable structured information to further support conclusions, diagnoses, and treatment decisions when the parent, teacher, and self-report scales are combined. The test contains 27 items and covers a subset of subscales namely the oppositional, cognitive problems or inattention, hyperactivity, and the ADHD (Attention Deficit Hyperactivity Disorder) index. According to the norms, scores above the 50^th^ percentile denote significant markers for behavioral problems in the specific domains. The internal consistency of the test is 0.86 and the test-retest reliability was found to be 0.85.

*The Connors Teacher Rating Scale (CTRS-3* ^rd^ *edition-short form)*

The short form for teachers contains 28 items. The scale should be used when time is of the essence and when multiple administrations over time are desired (26). The scales include the oppositional domain, cognitive problems or inattention, hyperactivity and the ADHD (Attention Deficit Hyperactivity Disorder) index. According to the norms, scores above the 50^th^ percentile denote significant markers for behavioral problems in the specific domains. The test-retest reliability was found to be 0.88 and the internal consistency of the test for each scale divided by gender ranged from 0.77 to 0.96.

*The Kinetic Family Drawing test (KFD)*

This is a projective test used to understand and assess the perspectives of children and adolescents on their families. According to one of the originators of the KFD, Robert C. Burns, the KFD allows us to see the self as it is reflected and expressed in the family. It enables the young person to depict the family as a functioning, active unit, and allows us to see the child's impressions of these dynamic interactions among family members (27). Interpretation of the KFD is based on the “projective hypothesis”, which is an assumption that an individual, when drawing a picture on a blank page, will project his or her thoughts, concerns, conflicts, needs, motivations, and frustrations into the picture. The examiner may then ask the child questions about the drawing, such as what is happening and who has been depicted in the picture. Certain characteristics of the drawing are noted upon analysis, such as the placement of family members, the absence of any members, whether the figures are relatively consistent with reality or altered by the child, the absence of particular body parts, erasures, elevated figures, and so on. Inferences about the picture depicted would be verified with parent perceptions and teacher reports to ensure objectivity. The therapist will have to interpret the drawing based on the colors used in the picture, spacing and how the child projects himself in it. Reliability scores for the test ranged from 0.65 to 1.00 with a median reliability of 0.87.

*The Child and Adolescent Sleep Checklist (CASC-student version)*

The CASC student version is designed to identify sleep habits and to make a screening of sleep problems among students 12-18 years of age (28). They are to complete the questionnaire themselves. The CASC scores are subdivided into four categories such as bedtime problems, unstable sleep, sleep movements and daytime problems. Responses are given a score of 3 for "always", 2 for "usually", 1 for "occasionally", or 0 for "never". Scores can range from 0-72. Children with CASC sleep disturbance score of 18 or more are considered to have sleep problems. The reliability and validity of the test was found to range between 0.8 and 0.98.

*The Adolescent Food Habit Checklist (AFHC)*

This test consists of questions that relate to the food habits of adolescent children between ages 13-16 years (29). The AFHC is usually administered to identify diet concerns and habits in children. It is a 23-item test that consists of true or false responses. A healthy response is given a score of 1 and the final AFHC score is obtained by multiplying the number of healthy responses by the product of the 23 items divided by the total number of responses in the test. According to the test, scores ≥ 9 indicate healthy eating habits for females and scores ≥ 11 indicate good eating habits for males. The internal consistency of the test was found to be 0.83 and the Cronbach’s alpha for the test is 0.91. 

*The Mood and Feelings Questionnaire (MFQ-Short form)*

This questionnaire contains a series of 13 phrases that can help describe how adolescents ages 13-18 have been feeling or acting recently (30). If the response for a sentence is "not true" it is given a score of 0, if the response is "sometimes" a score of 1 is given, and if the response is "true" a score of 2 is given. The total score of the child's mood is obtained by the sum of all the scores across the 13 items. A score ≥ 10 denotes that there are concerns in the child's mood. The internal reliability coefficient for the MFQ short form is 0.87. 

***Scheduled interviews***

The “interview” is a managed verbal exchange and as such its effectiveness heavily depends on the communication skills of the interviewer (31). These include the ability to clearly structure questions, pause, probe or prompt appropriately, and encourage the interviewee to talk freely (32). Interpersonal skills, such as the ability to establish rapport, perhaps with humor and humility, are also important. We need to understand that the relational aspect of trust is necessary between participants. Specially designed questionnaires could be used to gather information. In this study, we gathered information regarding the school, home, and peer environment. All questions were open-ended and non-confronting, and sessions during assessments were confidential (33). Parents were given opportunities to discuss difficulties they encountered, how they perceived the child’s ability, and what they intended to do about it. Interviews held with the child focused on his perceptions of the difficulties he experienced, what he expected from himself, and how he was going to make a difference. Debriefing was done every time a session was completed (34). The debriefing session is an important ethical consideration to make sure that participants are fully informed about, and not harmed in any way by their experience in the study. Parents were assured that assessment was not a diagnostic tool, but was done only to gather information about the child.

## Results

On the verbal tests of WISC IV, the child obtained a score of 70 indicating a “Borderline” intellectual level of functioning (Table 1). He had difficulties in the subtests of general information and arithmetic. On the performance tests, the child obtained a score of 84 indicating a “Dull normal” intellectual functioning. He executed most motor activities well and appeared to be motivated while performing them. He, however, had difficulty executing timed activities and required prompting to encourage him to finish the task. He had a good understanding of sequencing tasks and his visual spacing skills were adequate; however, he still needed improvement. Post intervention, the WISC IV, on repetition, showed an improvement on both verbal and performance tests. The child obtained a score of 73 for verbal quotient and 86 on the performance tests.

On the Vineland Social Maturity Scale (VSMS), the child obtained a social age score of 11 years (132 months) while his chronological age was 13 years (156 months). Social age was found to be age appropriate on domains such as eating, dressing, locomotion, and occupation. His social quotient was found to be 84 (Table 2). Problems were present in understanding social cues, communication, and in the self-direction domains. Post intervention, his social age improved from 11 to 12 years and the social quotient improved to a score of 88. Behaviorally the child appeared more confident and fulfilled.

On the Connors Parent Rating Scale (CPRS-short form), the child had low scores on hyperactivity, but scores on inattention and cognitive problems were observed to be greater than the 50^th^ percentile which denotes that these were significant behavioral problems (Table 3). He, however, did not fulfill the ADHD (Attention Deficit Hyperactivity Disorder) index. Post intervention, we observed an improvement as scores on the inattention and cognitive domains were found to fall below the 50^th^ percentile.

On the Connors Teacher Rating Scale (CTRS-short form), he had low scores on the oppositional behavior domain, but scores on the hyperactivity and inattention subscales were observed to be higher than the 50^th^ percentile which implies that this is a significant behavioral problem (Table 4). Post intervention, scores on hyperactivity and inattention had greatly improved and was found to fall below the 50^th^ percentile.

**Table 1:** Verbal and performance quotients pre and post intervention

	**Verbal quotient**	**Performance quotient**
**Pre intervention**	70	84
**Post intervention**	73	86

**Table 2:** Social age and social quotients pre and post intervention

	**Chronological age (CA)**	**Social age (SA)**	**Social quotient (SQ)**
**Pre intervention**	156	132	84
**Post intervention**	162	144	88

**Table 3:** Scores on each domain of the Connors Parent Rating Scale (CPRS) pre and post intervention

	**Oppositional behavior**	**Hyperactivity**	**Inattention**	**Cognitive problems**	**ADHD** † ** Index**
**Pre intervention**	> 25^th^ percentile and below 50^th^ percentile	> 25^th^ percentile and below 50^th^ percentile	> 50^th^ percentile	> 50^th^ percentile	> 25^th^ percentile and below 50^th^ percentile
**Post intervention**	> 25^th^ percentile and below 50^th^ percentile	> 25^th^ percentile and below 50^th^ percentile	> 25^th^ percentile and below 50^th^ percentile	> 25^th^ percentile and below 50^th^ percentile	> 25^th^ percentile and below 50^th^ percentile

† Attention Deficit Hyperactivity Disorder

**Table 4:** Scores on each domain of the Connors Teacher Rating Scale (CTRS) pre and post intervention

	**Oppositional behavior**	**Hyperactivity**	**Inattention**	**Cognitive problems**	**ADHD** † ** Index**
**Pre Intervention**	>25th percentile and below 50th percentile	>50th percentile	>50th percentile	>25th percentile and below 50th percentile	>25th percentile and below 50th percentile
**Post Intervention**	>25th percentile and below 50th percentile	>25th percentile and below 50th percentile	>25th percentile and below 50th percentile	>25th percentile and below 50th percentile	>25th percentile and below 50th percentile

† Attention Deficit Hyperactivity Disorder

**Table 5:** Scores and the interpretations on the Child and Adolescent Sleep Checklist (CASC), Adolescent Food Habit Checklist (AFHC), and The Mood and Feelings Questionnaire (MFQ) pre and post intervention

**Tests administered**	**Pre intervention** **scores**	**Post intervention** **scores**	**Interpretation**
**Child and Adolescent Sleep Checklist (CASC)**	10	8	Score is < 18; hence, "no difficulty" in sleep
**Adolescent Food Habit Checklist (AFHC)**	15	17	Score is > 11. Hence, this indicates "healthy" eating habits
**The Mood and Feelings Questionnaire (MFQ)**	6	5	Score is < 10; hence, "no difficulty" in mood

Interesting findings were revealed from analyzing the Kinetic Family Drawing (KFD) test. The child depicted no facial expression of himself or the parents. No bright colors were used and the drawing was very plain and dull. He seemed to be emotionally attached to his mother, since he drew his image closer to her. The father figure appeared larger and more prominent in the picture. Post intervention, the picture he drew of himself was a happy figure. He, however, had depicted himself in the corner, but there was an improvement in the emotions expressed in the picture.

On the Child and Adolescent Sleep Checklist (CASC), he obtained a score of 10 pre intervention and a score of 8 post intervention. Both these scores were lower than the cut off limit of 18 which implied that there were no concerns with sleep (Table 5). On the Adolescent Food Habit Checklist (AFHC), a score of 15 and 17 was observed pre and post intervention that implied no concerns in dietary patterns, since both these scores were well above the cut off limit of 11. Finally, a score of 6 and 5 was obtained on The Mood and Feelings Questionnaire (MFQ) pre and post intervention which was lower than the cut off limit of 10. This once again denoted that there were no concerns regarding the child’s mood or emotional regulation.

Scheduled interviews revealed that both parents were distressed. They had high expectations from the child and accepted that their child disappointed them. Societal pressures were evident in discussions. There were also hidden fears that parents had about the child’s future, the grades that the child needed to secure, and also about his occupation. They were pessimistic about how he would be able to perform in school and whether the authorities would be supportive enough.

Similar scheduled interview sessions with the child revealed that the child was also anxious. He expressed his concerns in adjusting in the classroom as he was unable to copy, write, or do arithmetic calculations in the pace that other children did. He especially felt singled out in class and was worried that the teacher was unable to give him the required individual attention. He felt misunderstood at home, since his parents were concerned only about his academic work and spared less time conversing with him. He said that the long tuitions hours caused more fatigue and disinterest in going to school. The child admitted that most of his childhood was being lost and that studying was becoming too cumbersome.

## Discussion

On the verbal tests of WISC-IV, the child obtained a score of 70. He had a good vocabulary, understood meanings of words, and used them in grammatically correct sentences. On the general information subtest, he did not know answers to questions such as “what is photosynthesis?” or “what is hieroglyphics?”, and therefore kept guessing and turning to his parents for help. His comprehension was adequate for his age as he was able to reason to questions such as “what would you do if you got lost?” or “what would you do if you lost your friend’s ball?”. He also answered questions on similarities between objects by relating to their sizes, shapes, and colors, and therefore had good association skills. His expressive language was good and he had no difficulties in pronunciation, but was slow in conservations regarding his school or hobbies. He found it difficult to narrate experiences spontaneously and he needed much probing to do so. He took a long time responding to pictures when shown to him and in explaining the sequence of events that he saw occurring in the picture. He required much encouragement to speak in a social setting. Numerical problems in the arithmetic subtest were easy at first, but when the complexity increased he could not calculate it mentally. He particularly found fractions difficult to understand and the concept of money hard to solve.

On the performance tests, the child obtained a score of 84 indicating a “Dull normal” intellectual functioning. On the first subtest of geometric design test, he had to arrange blocks as seen in the picture given. We observed that he had no difficulty in visual spacing or in organization skills, as he arranged the blocks according to the pattern. On the second subtest of picture completion, he was prompt in identifying and naming the missing parts in the picture. On the coding subtest, which was a writing activity, he represented numbers as symbols. According to studies, it has been found that children with learning disabilities experience problems in writing because of neurological defects that create difficulty in comprehending written language and this could also underlie problems in understanding nonverbal communication (35). Though we observed that his writing was slightly messy and slow, there were no letter reversals, omissions, or deletions while copying words or writing sentences. On the sequencing subtest, he had to arrange pictures in the correct order, but as items increased in their complexity, there was confusion that was clearly evident. He particularly found puzzles difficult to complete on the object assembly subtest. Post intervention, the WISC IV, on repetition, showed an improvement on both verbal and performance tests. The child obtained a score of 73 for the verbal quotient and 86 for the performance quotient. Though IQ levels cannot change over time, we still identified that there was observable improvement in significant areas. He was better with his vocabulary and expressed well in grammatically correct sentences. He engaged in conversations spontaneously and narrated experiences with ease. He showed improvements in understanding concepts of money and time in arithmetic. He also explained differences and similarities of objects better than his first attempt. On the performance tests, the speed of execution and dexterity had particularly improved.

On the Vineland Social Maturity Scale (VSMS), the child obtained a social age score of 11 years (132 months) while his chronological age was 13 years (156 months). His social quotient was found to be 84 and we observed that there is a delay of two years in his social skills. Though his social age was age appropriate on domains such as eating, dressing, locomotion, and occupation on the VSMS, problems were present in understanding social cues, communication, and in the self-direction domains. According to studies, children and adolescents with learning disabilities are found to be less sensitive to the social meanings of gestures and facial expressions and have more difficulty discriminating vocal tones (36, 37). This lack of sensitivity could seriously undermine social interactions in individuals with learning disability. Interpersonal problems in those with learning disability may be viewed as the consequence of an impaired ability to attain and apply metacognitive rules and strategies (38). Children with learning disability tended to produce less varied and more rigid coping strategies as they are unable to adapt appropriate cognitive strategies to different social situations (39). They also have difficulty in organizing spontaneous and efficient strategies that are directed to the achievement of social goals (40). Post intervention, there was an improvement in the scores of each domain. His social age improved from 11 to 12 years (144 months) and his social quotient was found to be 88. The child behaviorally appeared more confident and fulfilled. Having made provisions to reduce the burden of academic work, the family had been able to spend more quality time with the child and also plan for social opportunities. This had encouraged the child to engage in social interactions with peers and to also learn to respond to social cues appropriately.

On the Connors Parent Rating Scale (CPRS-Short Form), the child had low scores on the hyperactivity domain, but scores on inattention and cognitive problems were found to be greater than the 50^th^ percentile. He, however, did not fulfill the ADHD (Attention Deficit Hyperactivity Disorder) index. Parents admitted that the child found it difficult to sustain attention while learning and that he allowed himself to be distracted. They also mentioned that he did not complete academic work and that most often it is messy. According to them, they felt that the child had concerns in comprehending written information and that he took a longer time to read texts of information. Remembering what he had learnt was also difficult for the child. Post intervention, the Connors Parent Rating Scale (CPRS) revealed that he had lower scores on inattention and that scores on the cognitive subscale had also improved since they were below the 50^th^ percentile.

Teacher reports of classroom behavior were obtained by telephone conversations. According to many studies teacher reports were found to be highly reliable (41). Research has pointed out that children with learning disability achieve less peer acceptance, and therefore may have fewer opportunities to engage in social interactions, are usually quiet and shy in the class, and that they find it difficult to converse in a group and to accumulate social experiences that form the basis for interpersonal understanding (42-45). 

The Connors Teacher Rating Scale (CTRS-Short Form) revealed that he had low scores on the oppositional behavior domain, but scores on the hyperactivity and the inattention subscales were found to be greater than the 50^th^ percentile that denoted significant behavioral problems. The teacher reported that the child frequently daydreamed, appeared lost in class, and also distracted other children. Research has revealed that children with learning disabilities experience behavioral difficulties and problems such as aggression and misconduct that could occur in the classroom (46). Post intervention, the Connors Teacher Rating Scale (CTRS-Short Form) revealed that scores on hyperactivity and inattention had improved as they were below the 50^th^ percentile. Since parents had been consistent in using assistive techniques at home and school, the teacher reported that the child was taking an interest in academic work and that he appeared much more comfortable in class. Peer learning had also enabled him to build relationships with other children and to enjoy the experience of learning. Taking up small responsibilities at home and school had made the child feel proud of his abilities and improved his self-esteem.

The Kinetic Family Drawing test (KFD) pre intervention did not depict any happy facial expressions in either himself or his parents. When the child was asked to explain what he had drawn, he was reluctant and gazed away. According to studies, the most striking and significant fact was that in most of the studies that had used the KFD researchers observed no interaction whatsoever among family members; 80% of the mothers and 60% of the fathers were found to have a poor interaction with their child. Poor interactions are assumed to lead to a negative self-concept (47). 

No liveliness or excitement was depicted in the images, but the mother figure was closer to him. He said that he felt comfortable talking to her whenever he was troubled, but that his father always instructed him and demanded better grades every time he wrote his tests in class. The father figure depicted, was larger and more prominent occupying more space in the paper. He said that the father had hardly any time to spend with him, as he was always busy with work. 

Post intervention, the father figure that he drew was still prominent, but there was a positive emotion depicted in the picture. He had chosen better colors and much of the pessimism had gone away. When asked to explain, he seemed more comfortable and happier to say that his family was very important to him, that they were helping him do better in school and at home, and that he spent more time with them. He said that his father had got him interested in a few outdoor games and that every week he sets aside time to play with him. The father too, felt happier to make the effort to spend time with the child. According to studies, parents’ positive attitude towards their children and family support increases the child's confidence in their abilities and awakens the child’s interest in satisfying and meeting parents’ expectations (48).

There were no concerns on the Child and Adolescent Sleep Checklist (CASC), The Adolescent Food Habit Checklist (AFHC), and also on the Mood and Feelings Questionnaire (MFQ) pre and post intervention. He had healthy eating and sleeping habits, and his emotions were also stable. 

Scheduled interviews pre intervention, revealed that parents hesitated discussing much of their concerns, but gradually they had warmed up after a few sessions. There was reluctance in accepting that their child had a disability and when told that it was necessary to discuss with the teacher there was resistance. Studies have revealed that the birth of an exceptional child shatters many parental expectations (49). The typical stages such parents usually undergo are a loss of self-esteem, guilt, shame, depression, and ambivalence of contradictory, positive, and negative feelings toward the child (50). The entire family may sometimes turn into an exceptional family adopting a pattern of family seclusion (51). These behaviors may cause the child to forge a negative self-image, thereby damaging the development of his or her personality (52). 

Time and again the family was reassured that this was an evaluation done only to help the child and not to discriminate him from other children. Scores on the WISC IV and the VSMS were discussed with the parents and they were given time to ask their queries. They were helped to see the child’s strengths and to understand areas that he found difficult. Having understood the meaning of scores, parents were given time to set realistic objectives and to participate in planning the intervention. We designed an individualized education program keeping in mind the expectations of the parents, but having it made clear to them that improvement can be seen only as a gradual process and that input must be sustained and consistent. 

Post intervention, scheduled interviews were more of conversations, which were positive. Both parents were happy to have had the experience of teaching their son and being a part of the intervention program. They appeared to be more confident and the father especially had taken the effort to have personal conversations with the child to make him feel comfortable and supported. The mother had made the effort to establish a good rapport with the school authorities and the teacher had kept them informed of the intervention techniques used at school and the progress that the child was making. According to studies, it has been shown that parental involvement in education stimulates the child's motivation toward academic work, their commitment to school, and their perception of competence, control, and efficiency (53). It has also been found that parental involvement promotes children's correct academic development in general, and is therefore of special interest in the case of learning disability or attention deficit hyperactivity disorder (54). Thus, coordinated academic support between family and school, and an adequate level of family collaboration on academic work are factors that promote optimal learning. 

During the scheduled interviews with the child pre intervention, he appeared to be quiet and refused to talk much. He sometimes withdrew when asked about his ambitions and about his academics. According to previous studies, those with learning disabilities faced challenges in articulating that are often associated with low self-esteem, isolation, anxiety, and inadequate language skills. They are unresponsive in open questioning and have difficulty generalizing from experiences and thinking in abstract terms. This makes it difficult for them to share their experiences (55). He clearly did not pride himself on any ability that he had and was concerned that there was no time for extracurricular activities. He also felt that he had too few friends in school and the neighborhood. The family had only focused on his studies and marks and not provided him with adequate social experiences. Post intervention, his anxiety was beginning to fade away. He smiled more and engaged spontaneously in conversations. He did not ponder much when talked to and made pleasant eye contacts. He had begun to make friends in school and found it more comfortable to handle his academic work. He had learnt a few outdoor games and was actively involved in it. He spent time with the teacher and made efforts to complete the worksheets given. At home, he adopted a play method of learning arithmetic and improving his reading skills. He felt more confident while reading in front of his peers and when he was corrected he did not withdraw or feel shy. His grades had also improved and he appeared more at ease with himself. 

## Conclusion

Learning disability is neither a disease nor a disorder, but can be overcome with appropriate family and professional support. Early interventions and specially designed individual educational programs can make remarkable changes in the academic performance of the child. The professional team must bear in mind that involving the parents in designing and implementing the intervention plan plays a vital role in bringing about improvements. The primary focus must be on the strengths of the child and not on his inadequacies. Therapists need to help parents realize that they need to make the effort and take responsibility to guiding their child in his or her journey of self-discovery. The child must be provided adequate experiences to improve his social skills and to gain self-confidence. Assessments used in the clinic must provide objective and reliable information regarding the potential of the child and not be used as diagnostic labels. Teacher involvement also is of utmost importance. Their role is necessary in helping the child adapt to the demands of the school and not feel threatened. Authorities in the school need to be supportive and accommodative to the use of assistive technology that reduces study strain in children with special needs. Individualized education programs have to be tailored to suit the needs of the child and parents need to be active co-therapists in implementing the intervention at home and social settings. The profile of the child’s behavior, communication, and social skills will also have to be monitored carefully and periodically by the professional team and the teacher. Parents will have to be proactive and motivated. Therefore, helping a child with a learning disability is not by just finding solutions to the problem, but by continually supporting the family and the child in implementing it the right way. 

The implications of the present study is that, it had focused on an in-depth analysis of a child's cognitive, behavioral, and social functioning, and parents worked as active co-therapists in assisting and providing therapy. An effective intervention that focused on all the key problem areas was designed and the follow up was done after 6 months. All the tests were repeated post intervention to analyze the effectiveness of the treatment plan and the study used all the ethical considerations and did not disseminate confidential information. The limitation of the study is that it is a case analysis, and therefore we cannot generalize the findings. However, the strength of the study is that both qualitative and quantitative measures were used in collecting and tabulating information and the intervention which was provided proved to be an effective one.

## Footenote

**Declaration of interest:** None.
